# Long-term cognitive and autonomic effects of COVID-19 in young adults: a cross-sectional study at 28 months

**DOI:** 10.1080/07853890.2025.2453082

**Published:** 2025-01-16

**Authors:** Gopika Gopinath, Chinmay A. Suryavanshi, Pallavi L. C.

**Affiliations:** Department of Physiology, Kasturba Medical College Manipal, Manipal Academy of Higher Education, Manipal, Karnataka, India

**Keywords:** COVID-19, long-term effects, cognitive function, Heart rate variability, young adults

## Abstract

**Objectives:**

The COVID-19 pandemic, caused by SARS-CoV-2, has had profound global impacts since its emergence in late 2019. Whilst acute symptoms are well-documented, increasing evidence suggests long-term consequences extending beyond the acute phase. This study aimed to investigate the long-term cognitive and autonomic effects of COVID-19 in young adults.

**Materials and Methods:**

We conducted a cross-sectional study comparing young adults with a history of COVID-19 (*n* = 34) to matched controls (*n* = 34). Cognitive function was assessed using the Sternberg Task, Stroop Task, and Go/No-Go Task (GNG). Autonomic function was evaluated using heart rate variability (HRV) parameters.

**Results:**

The average time interval between COVID-19 infection and testing was 28.2 months. The COVID-19 group showed significantly increased reaction time in the 2-item absent condition (*p* = 0.044) and errors in the 4-item present condition (*p* = 0.012) of the Sternberg Task and increased neutral response time (*p* = 0.028) and the normalized time for completing the task (*p* = 0.022) in the Stroop Task. No significant differences were found in the GNG Task. HRV parameters did not differ significantly between groups, although trends toward higher overall HRV were observed in the COVID-19 group.

**Conclusion:**

Young adults who had COVID-19 infection approximately 28 months ago show minimal long-term impact on cognitive function and autonomic regulation. However, subtle cognitive inefficiencies persist, particularly in working memory and executive function tasks. These findings suggest a generally favorable long-term prognosis for young adults following mild to moderate COVID-19 but highlight the need for further investigation into persistent subtle cognitive effects and autonomic effects.

## KEY POINTS

- The first comprehensive assessment of cognitive function in young adults 28 months post COVID-19 reveals subtle, persistent inefficiencies in working memory and executive function, providing critical insights for long-term clinical management.
- No significant differences in HRV parameters suggest normalized autonomic function in young adults long after COVID-19 infection.
- Young adults show resilience to long-term cognitive and autonomic sequelae of COVID-19.

## Introduction

The COVID-19 pandemic, caused by the SARS-CoV-2 virus, has profoundly impacted global health, society and economies since its emergence in late 2019. Whilst the acute symptoms of COVID-19 are well-documented, mounting evidence suggests long-term consequences extending beyond the acute phase of illness [1–3]. The World Health Organization defines the post-COVID condition, also known as ‘long COVID’, as the persistence of symptoms for at least three months following SARS-CoV-2 infection that alternative diagnoses cannot explain [4]. Long-COVID is a multisystem condition that can occur irrespective of the initial severity of the disease [5]. Among the reported sequelae, increasing attention has been directed toward prolonged effects on cognitive function, even in individuals who experienced mild to moderate illness [2,6]. Although most research has focused on older adults or those who experienced severe COVID-19 requiring hospitalization, growing evidence suggests that even young adults with mild infections may experience persistent cognitive effects [7–10].

Recent systematic reviews and meta-analyses have reported a high prevalence of cognitive impairment in COVID-19 patients, with estimates ranging from 18% to 36% [11,12]. A systematic review by Fanshawe et al. found moderate impairments across multiple cognitive domains in COVID-19 patients, with executive function, learning and memory, and complex attention being the most frequently affected areas [11]. Similarly, Austin et al. conducted a meta-analysis focusing specifically on cognitive function following non-severe SARS-CoV-2 infection, finding small but significant deficits in executive function, attention and memory [13]. Some studies have suggested that cognitive impairments may improve over time [11,14,15], whilst others have found persistent deficits in some cognitive domains even one-year post-infection [7,8,10,16]. A recent investigation revealed the presence of persistent symptoms and subtle cognitive impairments among university students up to 39 months following their initial infection [9]. However, Francis and Thunell reported no significant cognitive impairments in college students who had previously been infected with COVID-19 [17]. The long-term persistence of these cognitive deficits, particularly in young adults who experienced mild infections and did not require hospitalization, remains uncertain.

Alongside cognitive changes, evidence suggests that COVID-19 may affect the autonomic nervous system [18,19]. Heart rate variability (HRV), a measure of the variation in time between successive heartbeats, serves as a noninvasive indicator of autonomic function [20]. Several studies have reported alterations in HRV parameters in COVID-19 patients and survivors, suggesting potential dysautonomia [21–23].

However, many of these studies have concentrated on the acute or early post-acute phase of COVID-19, typically within the first few months after infection and among an older age group [21,22]. There is a paucity of data on the long-term cognitive and autonomic effects of COVID-19, particularly in young adults who experienced mild to moderate illness. Moreover, the impact of vaccination on these potential long-term effects remains poorly understood.

The central autonomic network (CAN) plays a crucial role in regulating both cognitive processes and HRV [24]. Given the potential neuroinvasive properties of SARS-CoV-2 [5], investigating cognitive function and autonomic regulation in COVID-19 survivors is of particular interest. Therefore, this study aims to investigate the long-term effects of COVID-19 infection on cognitive function and heart rate variability in young adults. By focusing on a longer timeframe, we seek to elucidate whether any cognitive or autonomic alterations persist in otherwise healthy young adults well beyond the acute and post-acute phases of infection.

## Methods

Participants were recruited from the university student population between October 2023 and March 2024 using convenience sampling. Information about the study was disseminated through student networks and groups. The study comprised 68 participants aged 18–39 years, divided equally into two groups: those with a documented history of COVID-19 infection (*n* = 34, COVID-19 group) and those without any history of COVID-19 infection (*n* = 34, control group). For the COVID-19 group, infection had been confirmed by either RT-PCR or antigen testing at least 12 months prior to study commencement. Individuals were excluded if they had active symptoms of COVID-19 at the time of the study, had a history of hospitalization due to severe COVID-19, had any preexisting neurological, cardiopulmonary, visual, and auditory impairment, were taking any medications or were left-handed. The study was approved by Kasturba Medical College and Kasturba Hospital Institutional Ethics Committee (IEC clearance: 403/2023). This study was performed in line with the principles of the Declaration of Helsinki. All participants provided written informed consent before taking part in the study.

The study was conducted in a quiet room within the Physiology department, with regulated temperature, during the morning hours. Participants were advised to abstain from engaging in strenuous physical activity and consuming caffeinated food or beverages during the 24-hour period preceding the examination. Both the groups were administered a General Health Questionnaire-5 test (GHQ-5) to confirm the wellness of the participants at the time of the study. All participants had a score of ≤3. Edinburgh handedness inventory (EHI) was also done to confirm if their preferred hand was right. Participants of the COVID-19 group retrospectively reported all symptoms experienced during their SARS-CoV-2 infection, with symptom data collected through a standardized questionnaire.

*HRV:* HRV was recorded and analyzed according to the published standard guidelines using the Powerlab data acquisition system (PowerLab 4/25 T and Chart v5.4 Pro, AD Instruments, NZ) [25]. HRV was recorded with the participant lying in a supine position in a quiet room for 15 min. Subsequently, an artefact-free segment of 300 s of recording was selected for analysis. HRV data were analyzed using HRV Module for Chart v5.4 Pro, AD Instruments, NZ.

*Cognitive Tests:* Sternberg Task Test, Go/No-Go Task (GNG), and Color Stroop task were assessed using Psychology Experiment Building Language (PEBL) 2.0 software [26].

For STT, the participants were presented with two, four, or six letters. The subjects were instructed to memorize the letters. The sequence presented was repeated in the same sequence in the second round. The participants were asked to respond to the visual stimuli presented to them at a time, and they identified the stimuli as either present or absent within sequences of letters. A total of 300 trials were given per individual. This test measures short-term/working memory involving cognitive control processes, using the time recorded to respond to the stimuli and the errors made accordingly.

In the GNG task, the participants were presented with two different signals: for the ‘Go’ signal, the participant had to respond by pressing the right shift key, and for the ‘No-Go’ signal, the participant had to refrain from responding to the stimuli. The test contains 320 targets in two segments, with 160 targets each. The number of errors in each round & mean response time for target signals in each round were noted. This test measures sustained attention and inhibition.

The Color Stroop task measures a person’s selective attention capacity, processing speed, and interference and inhibitory control. The task consists of 120 words named in various colors but appeared on the screen in different colored letters in a random sequence. The participants were instructed to respond by pressing the corresponding color code and ignoring the word. The time taken to respond, and the number of errors were noted. Utilizing the time for completing the task (TCT) and the total errors made (ne), we determined the participant’s accuracy percentage (ACC) using the equation (168 − ne)/168 × 100, and the normalized time for completing the Stroop task (NTCT) was computed by the equation TCT × 168/(168 − ne) [27].

### Statistical analysis

Descriptive statistics were used to describe the demographic variables. The normality of data distribution was assessed using the Shapiro-Wilk test for Cognition function parameters and the Anderson-Darling normality test for HRV parameters. For normally distributed variables, independent samples t-tests were used to compare differences between the COVID-19 and healthy control groups. For non-normally distributed variables, Mann-Whitney U tests were employed. All data analysis was conducted with RStudio. The significance level for all analyses was set at *p* < 0.05.

#### Results

This study enrolled a total cohort of 68 participants, of whom 58.82% were female. Table 1 presents detailed demographic information for the participants. All parameters were comparable between groups, with the exception of age, which was statistically significantly higher in the COVID-19 group. Among the 34 participants in the COVID-19 group, the prevalence of symptoms at the time of infection was as follows: fever (82.35%), ageusia (61.76%), anosmia (61.76%), fatigue (67.65%), cough (47.05%), headache (44.12%) and back pain (35.29%). The mean duration between infection and the month in which tests were conducted was 28.17 months. Most participants received an average of two vaccine doses, with only one participant having received no doses and seven receiving up to three doses. The mean duration between the last dose of vaccine and the month of testing was approximately 23 months. Twenty-four subjects were infected prior to vaccination, whilst ten were infected post-vaccination.

**Table 1.** Demographic data of the participants.

Variables	Control group	COVID-19 group
Number of participants	34 (14 Males, 20 Females)	34 (14 Males, 20 Females)
Age (year)*	22.82 (± 2.72)	24.76 (± 4.49)
Education (No. of years)	15.32 (±1.7)	14.97 (± 0.9)
Height (cm)	167.06 (± 8.68)	165.27 (± 8.87)
Weight (Kg)	63.99 (± 15.22)	62.52 (± 13.07)
BMI (Kg/m^2^)	22.87 (± 5.23)	22.85 (± 4.22)
Time since infection	–	28.17(± 7.74)
Vaccination status		
0 dose	01	00
1 dose	01	01
2 doses	27	26
3 doses	05	07

Data are mean ± SD. BMI: Body mass index.

*Significantly different (*p* < 0.05).

#### Analysis for cognitive tests (Table 2)

**Figure 1.**
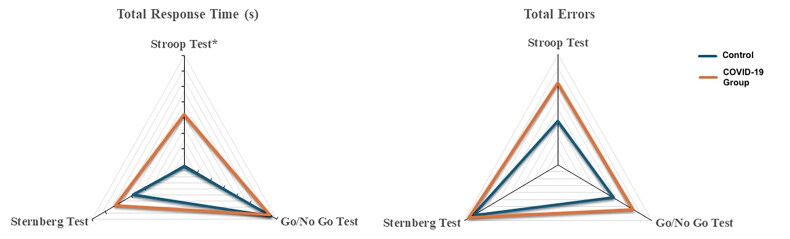
Total response time and total errors in various cognitive function tests in the two groups. *NTCT: normalized time for completing the Stroop task was significantly higher (*p* = 0.022) than the control group.

In the Stroop test, reaction times were slower in the COVID-19 group; however, only NTCT (*p* = 0.022) and NRT (*p* = 0.028) showed statistically significant differences (Figure 1). No significant between-group differences were observed for accuracy or error scores.

The Go/No-Go task parameters showed no significant differences between groups for response times, total errors, omission errors or commission errors (Supplementary Figure 1).

For the Sternberg task, significant differences were found only in the reaction time for the 2-item absent condition (*p* = 0.044) and errors in the 4-item present condition (*p* = 0.012), with the COVID-19 group performing worse than controls (Supplementary Table 1). No other parameters showed significant between-group differences (Supplementary Figures 2 and 3). In various cognitive tests, the COVID-19 group showed larger standard deviations in several cognitive parameters compared to the control group (Table 2).

**Table 2.** Cognitive function test parameters in the two groups.

Cognitive function tests and parameters assessed		Control Group (*n* = 34)	COVID-19 Group (*n* = 34)	*p* value
Stroop test	NTCT (s)	147.09 ± 23.42	160.88 ± 25.14	0.022*
	CRT (s)	0.743 ± 0.105	0.804 ± 0.137	0.051
	IRT (s)	0.886 ± 0.156	0.960 ± 0.166	0.059
	NRT (s)	0.786 ± 0.136	0.849 ± 0.133	0.028*
	Accuracy	96.24 ± 2.98	94.69 ± 5.00	0.139
	Total errors	6.32 ± 5.01	8.91 ± 8.41	0.139
	Congruent errors	1.62 ± 1.61	1.68 ± 1.96	0.766
	Incongruent errors	2.88 ± 2.38	4.85 ± 4.42	0.796
	Neutral errors	1.82 ± 1.96	2.38 ± 2.97	0.669
Go/No-Go task	Total response time (s)	319.4 ± 9.11	323.8 ± 11.59	0.101
	Total errors	9.44 ± 5.35	9.65 ± 5.65	0.878
	Commission errors	8.44 ± 4.77	8.03 ± 5.15	0.733
	Omission errors	1 ± 1.52	1.62 ± 2.87	0.904
Sternberg task test	Total errors	14.53 ± 9.31	11.4 ± 6.36	0.124
	Total response time (s)	249.3 ± 41.79	260.02 ± 42.4	0.146

Data are mean ± SD. NTCT: normalized time for completing the task; CRT: congruent response time; IRT: incongruent response time; NRT: neutral response time.

*p < 0.05.

**Table 3.** Heart rate variability measures in the three participant groups.

	Control Group (*n* = 34)	COVID-19 Group (*n* = 34)	*p* value
Heart rate (bpm)	76.15 ± 11.76	76.12 ± 10.03	0.991
SDNN index (ms)	57.73 ± 25.01	62.09 ± 18.56	0.416
Median RR (ms)	825.34 ± 128.28	846.35 ± 95.09	0.445
rMSSD (s)	53.94 ± 37.88	56.84 ± 28.84	0.443
pNN50 (%)	27.41 ± 22.9	28.39 ± 20.77	0.759
TP (ms²)	4177.04 ± 4338.94	4256.86 ± 2446.71	0.246
LF (nu)	41.75 ± 12.95	44.14 ± 17.64	0.526
HF (nu)	56.89 ± 12.28	54.28 ± 16.68	0.465
LF/HF	0.82 ± 0.432	1.05 ± 0.88	0.701

Data are mean ± SD. HR (bpm): resting heart rate in beats per minute (bpm); SDNN: Standard Deviation of Normal-to-Normal; rMSSD: root mean square difference of successive R-R interval; HF: high frequency (HF; 0.15–0.4 Hz); LF: low frequency (LF; 0.04–0.15 Hz); TP: total power; nu: normalized unit; ms: millisecond; ms2: milliseconds squared.

*Significantly different (*p* < 0.05).

#### Analysis for HRV (Table 3)

Analysis of HRV parameters revealed no statistically significant differences in either the time domain or frequency domain. However, the values for SDNN Index, Median RR, rMSSD, pNN50, TP, LF (nu), and LF/HF ratio were higher in the COVID-19 group.

## Discussion

This study aimed to investigate the long-term cognitive and autonomic effects of COVID-19 infection in young adults. The average duration between COVID-19 infection and testing was approximately 28 months. Our findings suggest that whilst some subtle differences persist, overall cognitive function and HRV remain largely unaffected in this population.

In the Sternberg Task, which evaluates working memory, we observed a statistically significant increase in reaction time for the 2-item absent condition and errors in the 4-item present condition in the COVID-19 group. This isolated finding may indicate subtle, persistent effects on working memory processes. However, the lack of significant differences in other Sternberg Task parameters suggests that overall working memory function is largely preserved. This partially corroborates results from Manna et al. who found selective impairment in auditory working memory but not visual working memory in 17 young adults 6–8 months after COVID-19 in a cross-sectional investigation [28]. Another cross-sectional study examining 319 patients of varying disease severity approximately 320 days post-infection revealed no significant differences in working memory performance when assessed *via* digit span testing [29]. Interestingly, the errors were lower in the COVID-19 group in the present study, though not statistically significant. This pattern could suggest a speed-accuracy tradeoff, where the COVID-19 group might be compensating for slower processing by being more careful in their responses [30]. A systematic review on working memory in 2023 by Cui et al. concluded that COVID-19 can cause a decline in working memory ability [31]; however, working memory scores gradually increased over a period of 17 months after COVID-19 [14].

The Stroop task revealed significant increases in NTCT and NRT for the COVID-19 group. Our findings are partially consistent with those of a cross-sectional study by Akinci et al. who found no significant differences in Stroop time or errors among 50 young adults with mild to moderate COVID-19 at 21 to 60 days following acute illness, although their experimental group performed more poorly on these tests compared with the control group [32]. Similarly to our findings, a cross-sectional study by Clemente et al. demonstrated increased response latency in the Stroop test amongst 32 older COVID-19 patients [33]. Although not statistically significant, we observed trends toward higher error rates (congruent, incongruent, and neutral errors) and lower accuracy in the Stroop task for the COVID-19 group. These trends, combined with the significantly increased NTCT and NRT, suggest a subtle cognitive inefficiency pattern that persists two years post-infection. This aligns with the findings of Fanshawe et al. who reported in their meta-analysis that executive function was one of the most frequently impaired cognitive domains in post-COVID patients [11].

Previous cross-sectional studies examining between 50 and 74 patients have demonstrated reduced performance in the GNG task amongst COVID-19 patients [34–36]. However, no statistically significant differences between the COVID-19 and control groups were found in our study. A longitudinal study by Diana et al. examining 22 patients observed an improvement in reaction times in a GNG task from 16 months to 22 months post-infection [15]. In a systematic review, a sub-analysis of studies having several time points demonstrated longitudinal enhancements in the cognitive domains of complex attention and memory. These improvements were most frequently observed 10–12 months subsequent to the initial COVID-19 infection [11]. It is important to note that whilst we found some significant differences and notable trends, many of our comparisons did not reach statistical significance. This could be due to several factors. Firstly, our study focused on young adults who had experienced mild to moderate COVID-19, a population that may be more resilient to long-term cognitive effects [9,10]. Secondly, the 28-month time frame since infection may have allowed for substantial cognitive recovery, as suggested by longitudinal studies showing improvement in cognitive function over time [14,15]. Also, a notable finding was the larger standard deviations in most of the cognitive test parameters in the COVID-19 group compared to controls, suggesting higher variations in the lingering effects of infection.

Importantly, our study found no significant differences in HRV parameters between the COVID-19 and control groups. Although not statistically significant, the trend toward higher SDNN Index, Median RR, rMSSD, pNN50, TP, LF, and LF/HF ratio in our COVID-19 group suggests a slight shift toward increased overall HRV. This trend aligns more closely with findings reported by Asarcikli et al. in their retrospective study of 60 patients [37]. It suggests that whilst reduced HRV is typically associated with COVID-19 infection, increased HRV may also be observed depending on the individual’s current inflammatory state and modulation by the PNS [38]. The current study finding aligns with the results of previous studies, which also found no differences in HRV measures in young adults with a history of COVID-19 [39,40]. Skow et al. conducted a cross-sectional study involving 27 young adults, while Freire et al. carried out an observational follow-up case-control study with 20 patients [39,40]. A recent longitudinal study involving repeated measures at six and thirteen months on 67 healthcare workers with mild COVID-19 demonstrated the resolution of autonomic cardiac regulation imbalance at thirteen months post-infection [41]. These consistent findings across studies suggest that autonomic function may be normalized in young, otherwise healthy adults over time, even after COVID-19 infection. The absence of significant HRV differences in our study contrasts with many other studies that have reported decreased HRV in COVID-19 individuals [22,42,43]. The longitudinal study by Adler and colleagues, involving 18 patients, demonstrated reduced HRV at three and six months following hospitalization for COVID-19 infection [42]. Similarly, a cross-sectional study by Haischer et al. reported reduced HRV in 41 COVID-19 survivors 7–8 months post-infection [38]. Similarly, another prospective study involving 103 patients found altered HRV, suggesting that autonomic dysregulation persists approximately 250 days after COVID-19 infection, which may predispose to cardiovascular complications [44].

The discrepancy with some previous studies may be attributed to the longer time elapsed since infection in our cohort, the younger age of our participants and the vaccination status of the participants. The average duration between infection and testing in our study was 28.2 months, which is considerably longer than most previous investigations. This extended timeframe may explain the limited cognitive and autonomic differences observed, as it allows for potential recovery and compensation mechanisms to take effect. Our findings support the notion proposed by Freire et al. that young adults may be more resilient to long-term autonomic and cognitive sequelae of COVID-19 [40]. The high vaccination rate of our participants may have played a protective role, potentially mitigating long-term effects. This is particularly interesting given that 24 subjects were infected before vaccination and 10 after, suggesting that vaccination may have protective effects even when administered post-infection. Sharma et al. suggested that vaccination might be beneficial in terms of being associated with less cardiac autonomic dysfunction, which could partly explain our findings [45]. However, the current cross-sectional study design does not allow for a direct assessment of vaccination’s impact on cognitive or autonomic outcomes. The potential protective effect of vaccination on long-term autonomic function of COVID-19 is an area that warrants further investigation.

The subtle differences observed in reaction times, particularly in the Stroop task, warrant further investigation. These findings may indicate that whilst overall cognitive performance is largely preserved, there might be underlying inefficiencies in information processing that persist long after the acute infection. This could have implications for demanding cognitive tasks or high-stress situations where rapid information processing is crucial. Limitations of our study include the relatively small sample size and the cross-sectional design, which precludes assessment of within-subject changes over time. Additionally, the lack of pre-infection baseline data limits our ability to detect subtle individual changes that may not be apparent in group comparisons. A significant limitation was the age disparity between groups, with the COVID-19 group being older than the control group. Future studies should consider longitudinal designs with larger sample sizes to better elucidate cognitive and autonomic function trajectory of COVID-19.

## Conclusion

The outcomes of this study suggest that young adults who experienced COVID-19 infection approximately two years ago show minimal lasting impact on cognitive function and autonomic regulation as measured by HRV. The isolated findings of increased reaction times in specific tasks indicate subtle, persistent effects and warrant further investigation but do not indicate widespread cognitive impairment. These results provide cautiously optimistic evidence for the long-term prognosis of young adults following COVID-19 infection. However, larger longitudinal studies are needed to confirm these findings and explore potential factors influencing recovery trajectories.

## Supplementary Material

Supplemental Material

## Data Availability

The data supporting the findings of this study are not publicly available to protect the privacy of research participants. The corresponding author will share the data upon reasonable request.
