# Supplementary material for: Long-term cognitive and autonomic effects of COVID-19 in young adults: a cross-sectional study at 28 months
Source: Ann Med. 2025 Jan 16;57(1):2453082. doi: 10.1080/07853890.2025.2453082 (PMC11749284; doi:10.1080/07853890.2025.2453082)
Supplement: Supplemental Material [file IANN_A_2453082_SM0129.docx]

Supplementary Table 01 – Additional data for the Go/No-Go Task and Sternberg Task test

| Parameters  *(Mean ± SD)* | | Control Group  *(n=34)* | COVID-19 Group (*n=34)* | p value |
| --- | --- | --- | --- | --- |
| Go/No-Go Task | **Total Correct** | 310.56 ± 5.35 | 310.35 ± 5.66 | 0.878 |
|  | **Commission RT-1** | 455.32 ± 42.56 | 467 ± 44.45 | 0.251 |
|  | **Commission RT-2** | 515.1 ± 31.98 | 526.26 ± 39.35 | 0.203 |
| Sternberg Task Test | **2 Present RT** | 696.1 ± 119.33 | 715.18 ± 122.13 | 0.461 |
|  | **2 Absent RT** | 780.82 ± 178. 78 | 836.29 ± 138.64 | **0.044*** |
|  | **4 Present RT** | 762.26 ± 109.1 | 787.38 ± 128.73 | 0.294 |
|  | **4 Absent RT** | 876.85 ± 173.1 | 905.1 ± 156.78 | 0.357 |
|  | **6 Present RT** | 900 ± 146.97 | 916.1± 191.85 | 0.777 |
|  | **6 Absent RT** | 971.15 ± 217.57 | 1061.97 ± 274.13 | 0.095 |
|  | **2 Present Errors** | 2 ± 1.6 | 2.03 ± 2.61 | 0.550 |
|  | **2 Absent Errors** | 1.47 ± 1.74 | 1.38 ± 1.18 | 0.716 |
|  | **4 Present Errors** | 3.09 ± 2.03 | 1.79 ± 1.8 | **0.012*** |
|  | **4 Absent Errors** | 1.56 ± 1.94 | 1.44 ± 1.52 | 0.759 |
|  | **6 Present Errors** | 3.88± 2.87 | 2.53± 2.69 | 0.472 |
|  | **6 Absent Errors** | 3.41± 2.48 | 1.50± 1.46 | 0.054 |

*Data are mean ± SD. *p<0.05. RT: response time.*

**Supplementary Figure 1.** *Response time and Total correct in the Go/No-Go Task.*


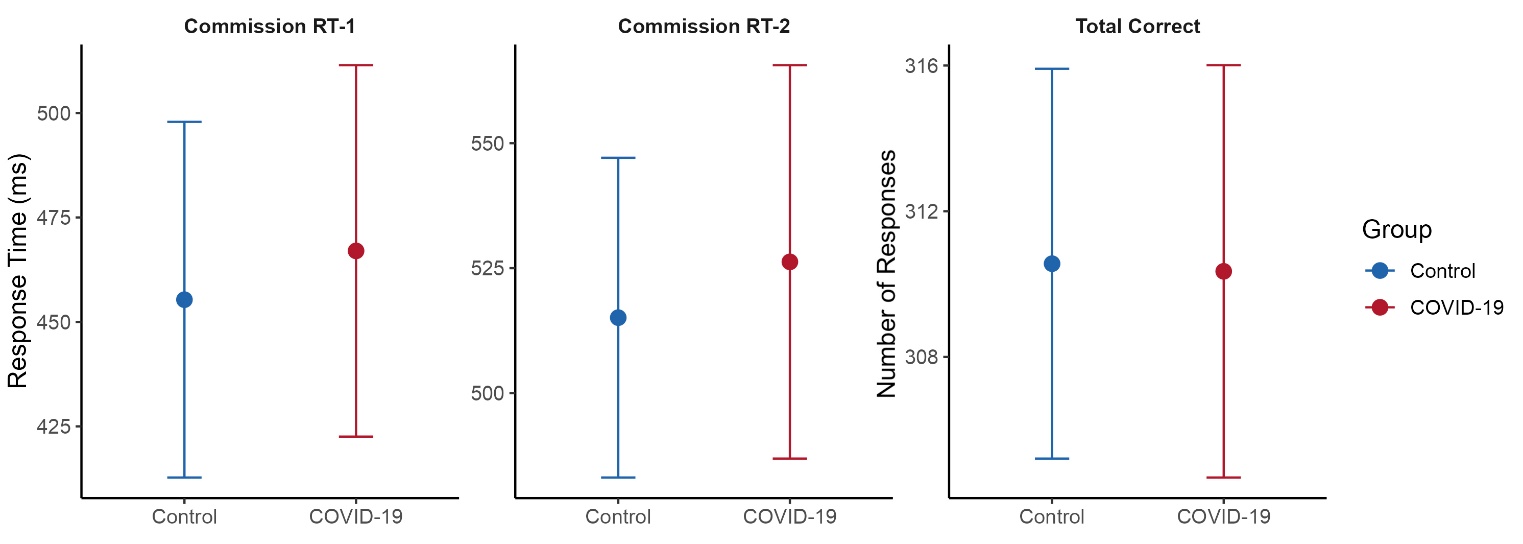


**p<0.05. RT: response time.*

**Supplementary Figure 2.** *Response time in the Sternberg Task test*

*
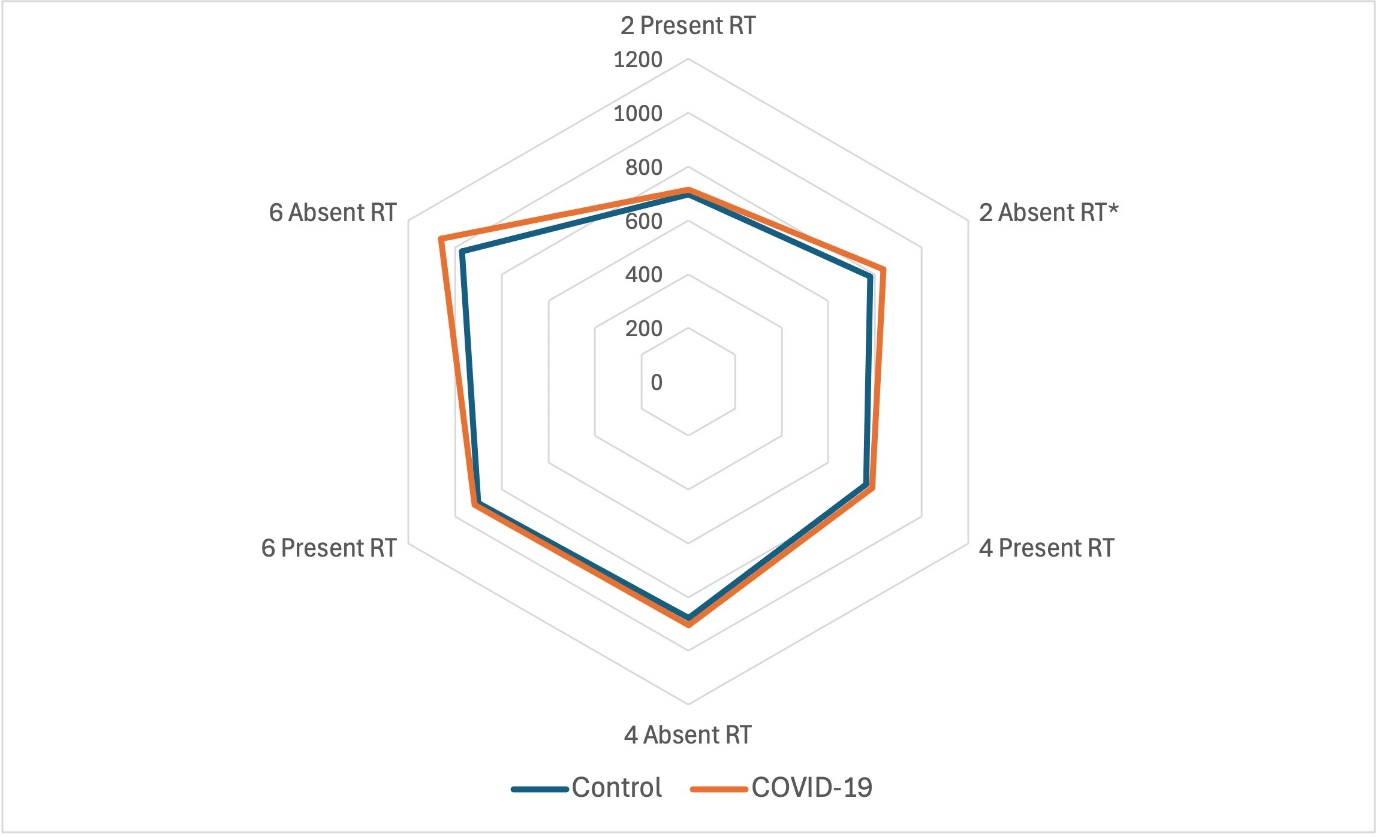
*

**p<0.05. RT: response time.*

**Supplementary Figure 3.** *Errors in the Sternberg Task test*

*
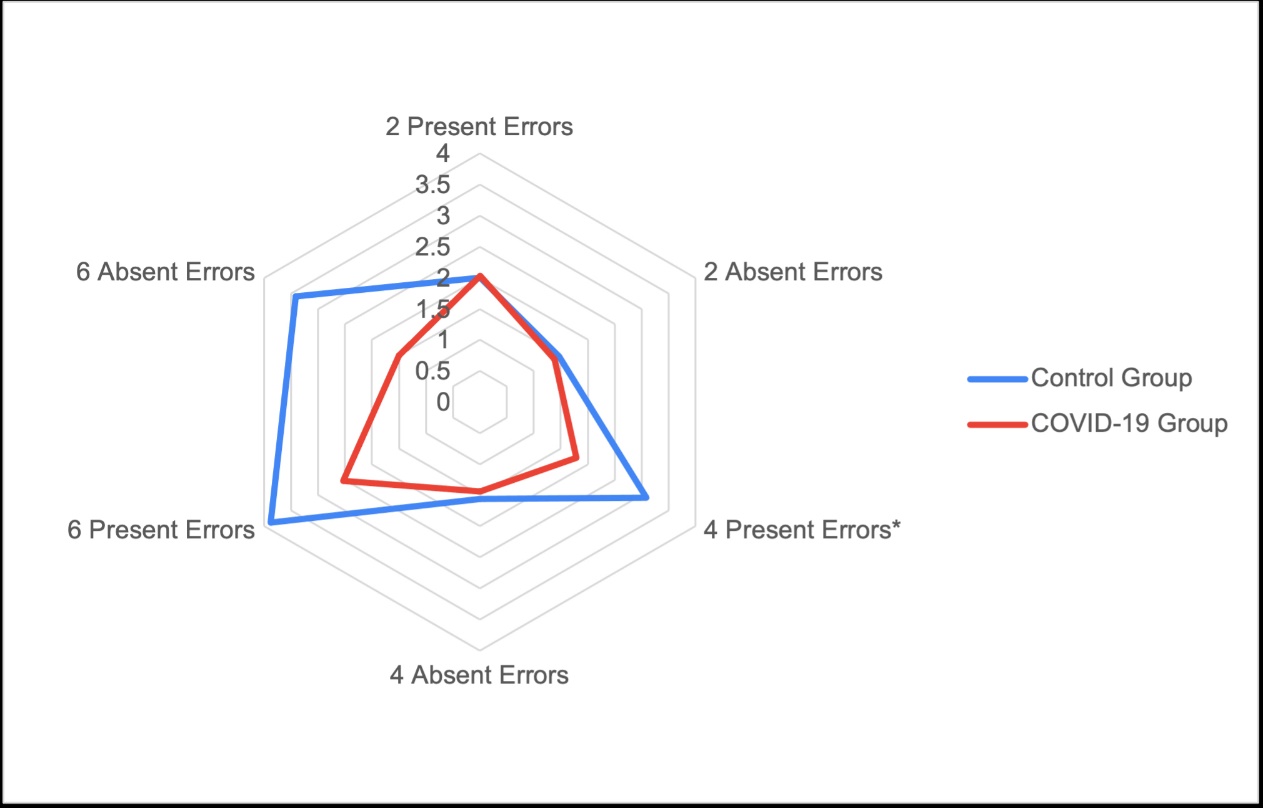
*

**p<0.05.*
